# 

**Published:** 1890-01

**Authors:** 


					﻿Enteredkmskmlnm
Vol. V.
JANUARY, 1889.
No. 7.
DANIEL'S
MEDICAL
JOURNAL
dnjnmkmlpjk,k;;;;;;;;;;;;;;;;;;;;;;;;;,iuhndmknml,l,;l
mskmwknml,mlm,l,
				

